# Supplementary material for: Can DyeCycling break the photobleaching limit in single-molecule FRET?
Source: Nano Res. 2022 May 13;15(11):9818–30. doi: 10.1007/s12274-022-4420-5 (PMC9101981; doi:10.1007/s12274-022-4420-5)
Supplement: Supplementary file 1 — Can DyeCycling break the photobleaching limit in single-molecule FRET? [file 12274_2022_4420_MOESM1_ESM.pdf]

Electronic Supplementary Material

Can DyeCycling break the photobleaching limit in single-molecule FRET?

Benjamin Vermeer, Sonja Schmid (✉)

NanoDynamicsLab, Laboratory of Biophysics, Wageningen University, Stippeneng 4, 6708WE Wageningen, The Netherlands

Supporting information to https://doi.org/10.1007/s12274-022-4420-5

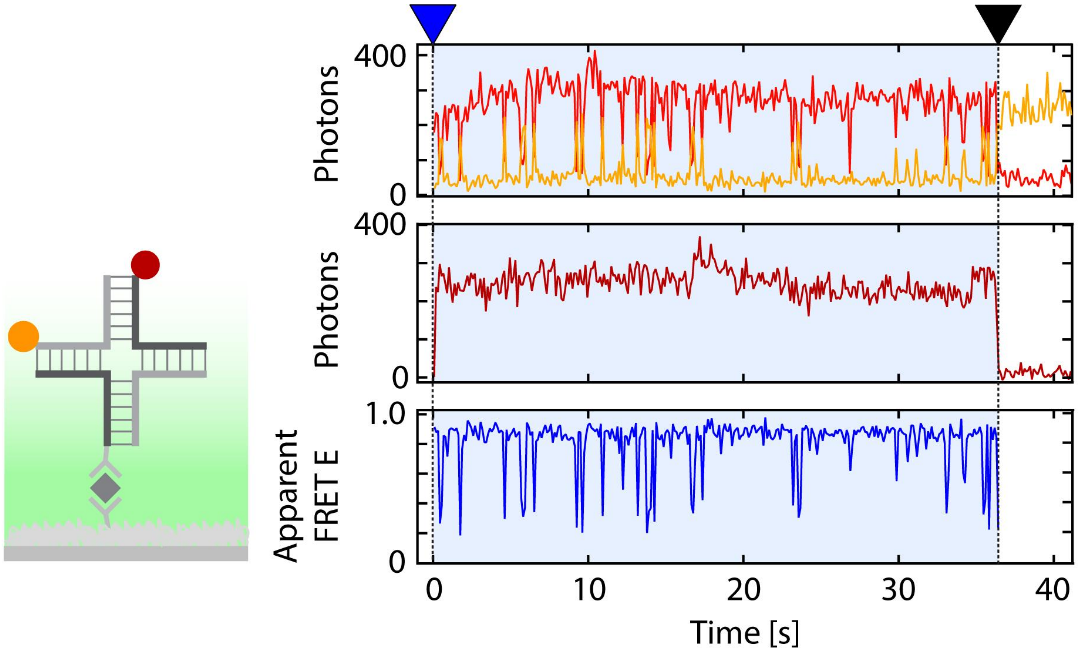

Figure S1 Covalently labeled Holliday junction under identical conditions as the DyeCycling measurement. Left: Illustration of a Holliday junction with a covalently attached donor (orange) and acceptor fluorophore (dark red). Right: Fluorescence and apparent FRET trajectories show the conformational changes of a Holliday junction as anticorrelated spikes in the top panel, and spikes in the bottom panel. Colors: donor: orange, FRET-sensitized acceptor: red, directly excited acceptor: dark red, apparent FRET efficiency (FRET E): blue. Blue triangle: start of the FRET regime (blue shading). Black triangle: acceptor photobleaching and end of the FRET regime.

Address correspondence to schmid@nanodynlab.org

Table S1 Holliday junction oligos and modifications

**Identifier**	**Sequence**	**Modification**
HJ1	5’-CCC TAG CAA GCC GCT GCT ACG G-3'	
HJ2	5’-CCG TAG CAG CGA GAG CGG TGG G-3'	
HJ3	5’-CCC ACC GCT CTT CTC AAC TGG GGA TTA TCG CCG TTC T-3'	5' biotin,<br>C6-amino linker
HJ4	5’-CCC AGT TGA GAG CTT GCT AGG GCA TTC TCC TGT G-3’	
DC1	5’-AGG AGA A**T**G-3'	dT-Atto647N,<br>C6-amino linker
DC2	5’-GCG ATA A**T**C-3'	dT-Atto550,<br>C6-amino linker
